# A primitive honey bee from the Middle Miocene deposits of southeastern Yunnan, China (Hymenoptera, Apidae)

**DOI:** 10.3897/zookeys.775.24909

**Published:** 2018-07-19

**Authors:** Michael S. Engel, Bo Wang, Abdulaziz S. Alqarni, Lin-Bo Jia, Tao Su, Zhe-kun Zhou, Torsten Wappler

**Affiliations:** 1 Division of Entomology, Natural History Museum, and Department of Ecology & Evolutionary Biology, 1501 Crestline Drive – Suite 140, University of Kansas, Lawrence, Kansas 66045-4415, USA; 2 Division of Invertebrate Zoology, American Museum of Natural History, Central Park West at 79th Street, New York, New York 10024-5192, USA; 3 State Key Laboratory of Palaeobiology and Stratigraphy, Center for Excellence in Life and Palaeoenvironment, Nanjing Institute of Geology and Palaeontology, Chinese Academy of Sciences, Nanjing 210008, China; 4 Key Laboratory of Zoological Systematics and Evolution, Institute of Zoology, Chinese Academy of Science, Beijing 100101, China; 5 Department of Plant Protection, College of Food and Agriculture Sciences, King Saud University, P.O. Box 2460, Riyadh 11451, Saudi Arabia; 6 Key Laboratory for Plant Diversity and Biogeography of East Asia, Kunming Institute of Botany, the Chinese Academy of Sciences, Kunming 650204, China; 7 Key Laboratory of Tropical Forest Ecology, Xishuangbanna Tropical Botanical Garden, the Chinese Academy of Sciences, Mengla 666303, China; 8 Natural History Department, Hessisches Landesmuseum Darmstadt, Friedensplatz 1, D-64283 Darmstadt, Germany

**Keywords:** Aculeata, Apinae, *Apis*, Apoidea, Miocene, taxonomy

## Abstract

While fossils of honey bees (Apini: *Apis* Linnaeus) are comparatively abundant in European Oligocene and Miocene deposits, the available material from Asia is scant and represented by only a handful of localities. It is therefore significant to report a new deposit with a fossil honey bee from southern China. Apis (Synapis) dalica Engel & Wappler, **sp. n.**, is described and figured from Middle Miocene sediments of Maguan County, southeastern Yunnan Province, China. This is the first fossil bee from the Cenozoic of southern China, and is distinguished from its close congeners present at the slightly older locality of Shanwang, Shandong in northeastern China. The species can be distinguished on the basis of wing venation differences from other Miocene *Apis*.

## Introduction

Honey bees (genus *Apis* Linnaeus) are iconic insects. The domesticated Western honey bee, *Apis mellifera* Linnaeus, is one of the most intensely studied animals (Winston 1991). Although most work focuses on *A. mellifera* for obvious apicultural and agricultural purposes, *A. cerana* Fabricius is also intensively managed and the remaining species are similarly exploited for their wax and honey. Honey bees comprise seven extant species of the corbiculate apine tribe Apini (Engel 1999a; Radloff et al. 2011), all of which are highly eusocial, with fixed queen and worker castes. This eusocial organization is shared with the related tribe Meliponini (stingless bees), while bumble bees (Bombini) occupy the primitively eusocial behavioral grade (Michener 1974, 2007). The putatively basalmost tribe of corbiculate bees, the Euglossini (orchid bees), are solitary or communal, with a few examples of primitive eusocial behavior in some species (Boff et al. 2015; Andrade et al. 2016). Relationships among these tribes have been controversial, although most evidence converges on a Darwinian null-hypothesis supporting a single origin of eusociality in the common ancestor of Bombini + Meliponini + Apini, and a single origin of the highly eusocial grade in the common ancestor of Meliponini + Apini (Michener 1990; Schultz et al. 1999, 2001; Engel 2001a; Noll 2002; Cardinal and Packer 2007; Canevazzi and Noll 2015; Porto et al. 2016, in press). Alternatively, some molecular evidence has placed meliponines as sister to bombines (e.g., Cameron and Mardulyn 2001; Kawakita et al. 2008; Rodriguez-Serrano et al. 2012), although in the most recent such analysis data from Euglossini were excluded (Kwong et al. 2017), and the potential impact of excluding one of the four surviving corbiculate tribes for driving spurious results has not been explored.

As is the case for most bees, the fossil record of corbiculate Apinae is comparatively sparse and largely confined to the Cenozoic, with a heavy bias toward material of Eocene through Miocene ages (Zeuner and Manning 1976; Engel 2001b, 2005; Ohl and Engel 2007; Michez et al. 2012). Euglossini have a meagre record, confined to the Early Miocene (Burdigalian) and younger deposits (Engel 1999b, 2014; Hinojosa-Díaz and Engel 2007), although an enigmatic and difficult to interpret compression from the latest Eocene of North America could represent a stem-group euglossine (Dehon et al. 2014). Bombini have a slightly stronger record (Rasnitsyn and Michener 1991; Michez et al. 2012; Wappler et al. 2012; Prokop et al. 2017), which is in need of revision but demonstrates the persistence of the crown group since at least the Eocene. Perhaps owing to the fact that all species are highly eusocial, often with large numbers of individuals within perennial colonies, fossils of Meliponini and Apini are the most abundant. In fact, in sheer numbers meliponine fossils outpace those of all other bees combined, although this is entirely due to a preponderance of material of workers from one species, *Proplebeia dominicana* (Wille & Chandler), from the Early Miocene of the Dominican Republic (Camargo et al. 2000). All other fossil stingless bee species are rare, but span from the end of the Cretaceous (Maastrichtian) to Pleistocene copals (Michener 1982; Michener and Grimaldi 1988; Engel 2001b; Greco et al. 2011; Engel and Michener 2013a, 2013b). Honey bees, again largely based on fossils of the worker caste, are known from a sparse number of deposits (Zeuner and Manning 1976; Nel et al. 1999), but at some they can be found in large numbers (e.g., Armbruster 1938; Kotthoff et al. 2011). These fossils span a range of ages from the earliest Oligocene through to the Pleistocene (Engel 1998a, 1999a, 2006; Engel et al. 2009; Kotthoff et al. 2011), although the taxonomic status of several putative species remains to be evaluated. Aside from these tribes, three other corbiculate tribes were once present – Electrobombini, Electrapini, and Melikertini (Engel 1998b, 2001b; Wappler and Engel 2003; Patiny et al. 2007; Engel et al. 2013, 2014). These extinct tribes were all eusocial, with the latter two belonging to the highly eusocial clade (Engel 2000b, 2001a, 2001b), and for at least one there is relatively detailed information on pollen collection for populations from the Lutetian of Germany (Wappler et al. 2015; Grímsson et al. 2017). More extensive work is needed regarding the refinement of relationships, but it is possible that one group of electrapines, genus *Thaumastobombus* Engel, was more closely related to honey bees owing to the presence of a barbed sting (Engel 2001).

Among the fossil Apini, there is apparently a gradation of taxa leading from the earliest Oligocene to the Miocene appearance of the first species of the clade comprising the surviving subgenera *Micrapis* Ashmead, *Megapis* Ashmead, and *Apis* s. str. (Engel 1998a, 1999, 2006). The extant clades form a monophyletic group relative to earlier species, the subgenera *Priorapis* Engel, *Synapis* Cockerell, and *Cascapis* Engel composing a basal grade (Engel 1998a, 1999, 2006). While most of the fossil species are found across Eurasia, well within the bounds of the modern, native distribution of *Apis* in Europe, Africa, and Asia, at least one species occurred within western North America during the Middle Miocene (Engel et al. 2009; Kotthoff et al. 2013). Within Asia there are few localities with sufficiently preserved material of honey bees (e.g., Stauffer 1979; Hong 1983; Zhang 1989, 1990; Engel 2006), most specimens deriving from the Upper Miocene deposits of Shanwang in northeastern China (Hong 1983; Zhang 1989, 1990). Herein we report the finding of a new fossil honey bee species from the Middle Miocene deposits of southern China. The species belongs to *Synapis*, expanding not only the paleogeographic distribution of this group but extending their temporal presence slightly later into the Miocene, approximately 1–2 million years younger than those records from the Northeast.

## Materials and methods

**Figures 1–3.**
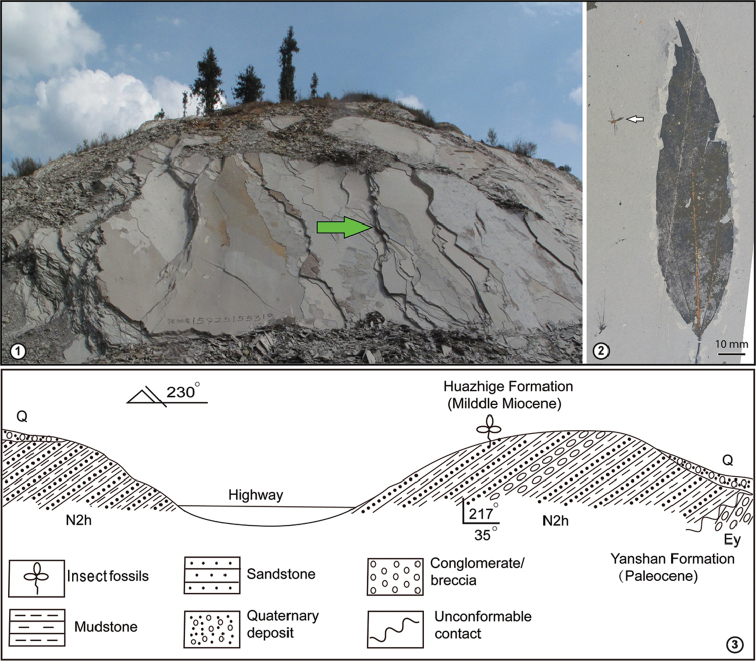
Fossil locality in Maguan County, southeastern Yunnan Province, China. **1** Outcrop overview, green arrow showing layers bearing the present fossil **2** Example of preservation, Acer cf. coriaceifolia H. Lév. (Sapindaceae) preserved together with a nematoceran fly (position indicated by white arrow) **3** Schematic cross section of the studied area.

Insect fossils were collected from the northwestern Maguan Basin, southeastern Yunnan, southwestern China (23°01'N, 104°23'E, 1320 m a.s.l.) (Figure 1). The Cenozoic sediments in Maguan are composed of the Paleogene Yanshan Group, Neogene Huazhige Formation, and Quaternary deposits (Zhang 1976; Bureau of Geology and Mineral Resources 1990; Zhang et al. 2015b). The basal Paleogene Yanshan Group is characterized by coarse breccias and lacks fossils (Zhang 1976; Zheng et al. 1999). Sitting unconformably on the Paleogene deposits, the Huazhige Formation is a ﬂuvio-lacustrine deposit, composed of light-gray or light-yellow pelitic laminated siltstone and mudstone, and bears abundant animal and plant fossils (Figure 2) (Zhang 1976; Zhang et al. 2015b). The Quaternary deposits overly unconformably on the Huazhige Formation (Zhang 1976; Zhang et al. 2015b).

The sediments bearing the present insect fossils are characterized by cyclic deposits of light-yellow or light-grey pelitic laminated mudstone and siltstone (Figure 3). They belong to the Huazhige Formation according to stratigraphic correlations (Zhang 1976; Zhang et al. 2015b). The Huazhige Formation is also well developed in the Wenshan Basin approximately 50 km to the north of the Maguan Basin, and the two basins are inferred to be the same age (Bureau of Geology and Mineral Resources 1990; Lebreton-Anberrée et al. 2016). The age of the Huazhige Formation in the Wenshan Basin was assigned to 16.5–15.2 Ma based on a recent palaeomagnetic study (Lebreton-Anberrée et al. 2016). Therefore, the age of the Huazhige Formation in the Maguan Basin should also be the Middle Miocene.

Besides insect fossils, the sediments bear abundant fossils of fishes, birds, as well as plants in excellent preservation (Figure 2). A preliminary study of plant fossils from the outcrop shows that the plant flora was dominated by Fagaceae and Fabaceae, accompanied by other elements such as *Calocedrus* Kurz (Zhang et al. 2015a), *Sequioa* Endl. (Cupressaceae) (Zhang et al. 2015b), *Bauhinia* L. (Fabaceae), *Burretiodendron* Rehder (Malvaceae) (Lebreton-Anberrée et al. 2015), *Cedrelospermum* Saporta (Ulmaceae) (Jia et al. 2015), and *Ailanthus* Desf. (Simaroubaceae), indicating a subtropical evergreen forest with warm and wet environment.

For the description, morphological terminology is adapted from Engel (2001b) and Michener (2007), with formats following previous studies on fossil honey bees (e.g., Engel 2006; Engel et al. 2009) and presented in the context of furthering refinements of species-level diagnoses for bees (e.g., Engel 2011; Gonzalez et al. 2013). The fossil is carbonized and so the integumental coloration or even patterning of lighter versus darker areas is not preserved. Photographs were taken using a Zeiss Stereo Discovery V16 microscope system at the State Key Laboratory of Palaeobiology and Stratigraphy, Nanjing Institute of Geology and Palaeontology, Chinese Academy of Sciences.

## Systematic paleontology

### Tribe Apini Latreille, 1802

#### Genus *Apis* Linnaeus, 1758

##### Subgenus Synapis Cockerell, 1907

###### Apis (Synapis) dalica

Taxon classificationAnimaliaHymenopteraApidae

Engel & Wappler sp. n.

http://zoobank.org/865F24F0-8027-4C7B-9B52-68116485FBDA

Figs 4–7 , 8–9

####### Holotype.

**Figures 4–7.**
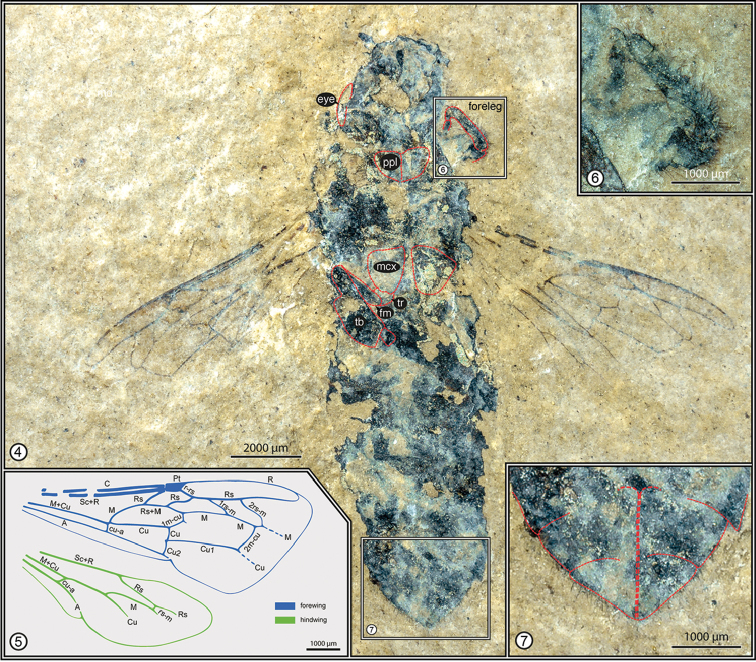
Holotype worker of Apis (Synapis) dalica Engel and Wappler, sp. n., from Maguan County, southeastern Yunnan Province, China. **4** Entire holotype (NIGP154200) as preserved **5** Reconstruction of wing venation; forewing above, hind wing below **6** Detail of foreleg. **7** Detail of apical sterna. Abbreviations: ppl = propleuron, mcx = mesocoxa, tr = trochanter, fm = femur, tb = tibia.

Worker (Figure 4), NIGP154200; Middle Miocene, approximately 16.5–15.2 Ma (around the Tortonian-Serravallian boundary); northeastern suburb of Maguan, Maguan County, Wenshan Zhuang & Miao Autonomous Prefecture, Yunnan Province, China. The holotype is deposited in the Nanjing Institute of Geology and Palaeontology, Chinese Academy of Sciences, Nanjing, China.

####### Diagnosis.

The new species is most similar to those Miocene honey bees described from Shandong Province, China. *Apis dalica* differs from them in the gently arched basal vein (comparatively straight in the specimens from Shandong), which is also closer to 1cu-a (separated by about a vein width versus several vein widths and even up to 0.5–0.75 times crossvein length in material from Shandong: refer to figures presented by Zhang 1989, and Zhang et al. 1994). In addition, in *A. longitibia* Zhang and *A. miocenica* Hong 2rs-m is comparatively straight (Zhang 1989; Zhang et al. 1994), rather than the distinctly arcuate form of *A. dalica*. In *A. shandongica* Zhang and *A. miocenica* 1m-cu is not so prominently arched and only so at its anterior end rather than strongly so and at midlength in *A. dalica*. Lastly, in all of the material from Shandong (Zhang 1989; Zhang et al. 1994), 1Rs originates in a strongly proximal position relative to the base of the pterostigma, rather than near the base of the pterostigma in *A. dalica*. The pterostigma of *A. dalica* is more distinctly developed than in modern species and most other fossil species of *Apis*.

####### Description.

Worker. Total length (as preserved) 17.06 mm; preserved in ventral orientation, with head thrust forward, wings extended obliquely away from body, and legs largely tucked underneath the body with most podites not preserved or indiscernible; coloration not preserved (appearing uniformly charcoal black). Head apparently slightly longer than wide as interpreted in ventral position; malar space elongate, longer than basal mandibular width; head narrower than mesosoma. Leg podites incompletely preserved. Metasoma typical for worker honey bee, length (as preserved) 9.03 mm, maximum width 4.36 mm; apical margins of sterna somewhat concave, those more basal sterna relatively straight, apical most sterna more strongly concave; sting not extended but slightly evident extending along midline of apical sterna (Figure 7).

**Figures 8–9.**
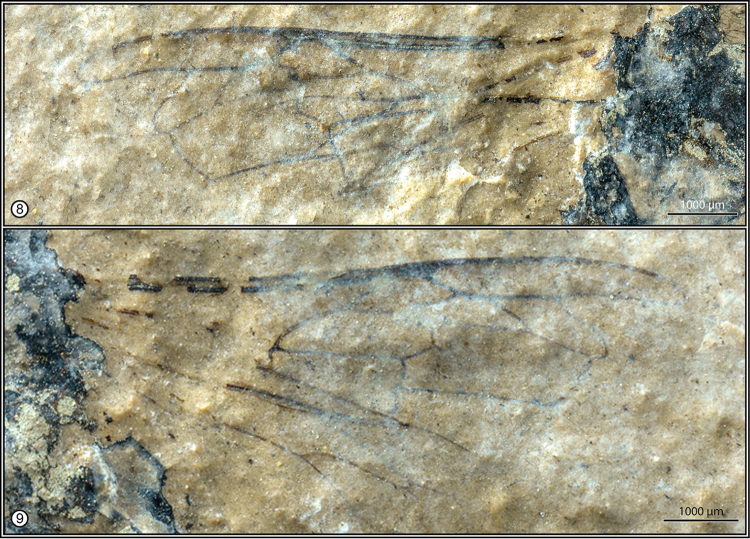
Wings of Apis (Synapis) dalica Engel and Wappler, sp. n., from Maguan County, southeastern Yunnan Province, China. **8** Details of right forewing **9** Details of left forewing.

Forewing with venation typical of *Apis* and subgenus Synapis (Figs 4, 5, 8, 9), length 8.54 mm, maximum width 2.18 mm; basal vein (1M) slightly distad 1cu-a, separated from 1cu-a by distance scarcely greater than vein width, gently arched before meeting 1Rs; 1Rs about as long as 1Rs+M and not in line with 1M; first submarginal cell smallest, with 2Rs sinuate (rather than relatively straight); r-rs about as long as anterior margin of second submarginal cell; second submarginal cell trapezoidal, with 1rs-m relatively straight and strongly slanted apically such that posterior border of cell is slightly more than three times length of anterior border; 1m-cu meeting posterior border of second submarginal cell at basal third of cell length, with distinct abscissal stub present at about angle of midlength, stub projecting into proximal border of second medial cell; third submarginal cell relatively broad anteriorly, with 2rs-m arcuate, anterior border of third submarginal cell distinctly longer than anterior border of second submarginal cell; aRs_2_ absent (sensu Tan et al. 2008); 2m-cu meeting posterior border of third submarginal cell near apical quarter of cell length, crossvein relatively straight. Hind wing with typical *Apis* venation, length 6.37 mm, maximum width 1.39 mm; linear series of distal hamuli present along anterior wing margin (precise number not discernible); distal abscissa M (‘indica’ vein) present, about as long as rs-m (Figure 5).

####### Etymology.

The specific epithet refers to the Medieval Dali Kingdom which occupied the area of Yunnan from its founding in 937 AD at the close of the Nanzhao Kingdom and until its termination by Kublai Khan (1215–1294) and the Mongol invasion in 1253 AD.

## Discussion

Fossil honey bees are comparatively uncommon in Asia relative to the wealth of material available from a variety of European deposits of Oligocene and Miocene ages (e.g., Nel et al. 1999; Kotthoff et al. 2011, 2013). In fact, most fossil honey bees in Asia have been found at a single locality in Shandong Province (Zhang 1989; Zhang et al. 1994). Unfortunately, the descriptions and available photographs of the material from Shandong are incomplete and there is reason to believe that some of the species from these deposits are synonyms of each other (Engel 1998, 1999), particularly in light of the fact that species of *Apis* can be notoriously variable in many features (e.g., Ruttner 1988; Radloff et al. 2010; Kotthoff et al. 2011, 2013). Thus, the present dearth of abundant specimens from which to work hampers a more comprehensive understanding of apine diversity in Asia during the Neogene, a period of time in which considerable diversification was apparently underway among honey bees such that by the present day the greatest number of species of *Apis* may be found across the Indomalayan region (e.g., Engel 1999, 2012; Michener 2007; Radloff et al. 2011).

The discovery of *A. dalica* expands the known localities with fossil honey bees southward in China and the presence of highly eusocial bees and critical pollinators within the Miocene of fauna of Yunnan. It is hoped that further exploration will recover larger numbers of workers from which the general morphometrics of the species can be determined and more precisely circumscribe the taxon among other Apini, as well as refine phylogenetic relationships among early honey bees. Phylogenetic studies on the modern species have demonstrated that open-nesting is ancestral for the genus (Engel and Schultz 1997). Given that most of the known fossil *Apis* fall basal to the clade of modern subgenera (e.g., Engel 2006; Kotthoff et al. 2013), and that *A. dalica*’s wing venation places it among species of the extinct subgenus Synapis, it is presumed that *A. dalica* would have constructed their nests in exposed localities, perhaps attached to the branches of trees or sturdy bushes. Such perennial colonies would have been more impacted by temperature changes over the course of the year, implying that the local paleoclimate was comparatively steady.

## Supplementary Material

XML Treatment for Apis (Synapis) dalica
